# The Interplay of Apoes with Syndecans in Influencing Key Cellular Events of Amyloid Pathology

**DOI:** 10.3390/ijms22137070

**Published:** 2021-06-30

**Authors:** Anett Hudák, Katalin Jósvay, Ildikó Domonkos, Annamária Letoha, László Szilák, Tamás Letoha

**Affiliations:** 1 Pharmacoidea Ltd., H-6726 Szeged, Hungary; anett.hudak@pharmacoidea.eu (A.H.); laszlo.szilak@gmail.com (L.S.); 2 Institute of Biochemistry, Biological Research Centre, H-6726 Szeged, Hungary; josvayk@brc.hu; 3 Institute of Plant Biology, Biological Research Centre, H-6726 Szeged, Hungary; domonkos.ildiko@brc.hu; 4 Department of Medicine, Albert Szent-Györgyi Clinical Center, Faculty of Medicine, University of Szeged, H-6725 Szeged, Hungary; letohadr@gmail.com

**Keywords:** ApoE, amyloid beta, syndecans, protein aggregation, endocytosis, neurodegeneration

## Abstract

Apolipoprotein E (ApoE) isoforms exert intricate effects on cellular physiology beyond lipid transport and metabolism. ApoEs influence the onset of Alzheimer’s disease (AD) in an isoform-dependent manner: ApoE4 increases AD risk, while ApoE2 decreases it. Previously we demonstrated that syndecans, a transmembrane proteoglycan family with increased expression in AD, trigger the aggregation and modulate the cellular uptake of amyloid beta (Aβ). Utilizing our previously established syndecan-overexpressing cellular assays, we now explore how the interplay of ApoEs with syndecans contributes to key events, namely uptake and aggregation, in Aβ pathology. The interaction of ApoEs with syndecans indicates isoform-specific characteristics arising beyond the frequently studied ApoE–heparan sulfate interactions. Syndecans, and among them the neuronal syndecan-3, increased the cellular uptake of ApoEs, especially ApoE2 and ApoE3, while ApoEs exerted opposing effects on syndecan-3-mediated Aβ uptake and aggregation. ApoE2 increased the cellular internalization of monomeric Aβ, hence preventing its extracellular aggregation, while ApoE4 decreased it, thus helping the buildup of extracellular plaques. The contrary effects of ApoE2 and ApoE4 remained once Aβ aggregated: while ApoE2 reduced the uptake of Aβ aggregates, ApoE4 facilitated it. Fibrillation studies also revealed ApoE4′s tendency to form fibrillar aggregates. Our results uncover yet unknown details of ApoE cellular biology and deepen our molecular understanding of the ApoE-dependent mechanism of Aβ pathology.

## 1. Introduction

As a 34 kDa glycoprotein constituent of plasma lipoproteins, apolipoprotein E (ApoE) facilitates the cellular transport and metabolism of lipids in the human body [1,2,3]. ApoE is expressed by several cell types, with the highest expression in the liver and the CNS [2,4,5]. Associated with almost all lipoprotein particles, ApoE mediates the clearance of lipoprotein remnants from the plasma via binding to specific cell surface receptors, including the LDL receptor family members and heparan sulfate proteoglycans (HSPGs) [6,7]. The 299-amino acid-long ApoE has two structural and functional domains: a 22 kDa NH2-terminal domain (amino acids 1–191) and a 10 kDa COOH-terminal domain (residues 216–299) containing the HSPG-binding region (residues 140–150) colocalized with the LDL receptor binding site [3,8]. ApoE exists in three isoforms that differ by only two amino acids: ApoE2 has cysteines located at sites 112 and 158, ApoE3 has a cysteine at site 112 and an arginine at site 158, while ApoE4 has arginines at both sites [5,9]. The cellular transport of lipoproteins is initiated by binding ApoE to the cell surface HSPGs, followed by their internalization mediated either by the LDL receptor or LDL receptor-related protein (LRP) or by the HSPGs alone [8,10,11,12].

Due to their highly polyanionic and versatile heparan sulfate (HS) glycosaminoglycan (GAG) chains, the heavily glycosylated cell surface HSPGs serve as binding sites for many proteins [13,14]. Membrane HSPGs contain two families: the transmembrane syndecans (SDCs) and glycosylphosphatidylinositol-anchored glypicans (GPCs) [13,14,15]. Interacting with a comprehensive network of ligands, including ApoE [13], membrane HSPGs exert wide-ranging biological activities, influencing cellular metabolism, transport, and signaling [13,16,17,18,19]. The structural diversity of HSPGs’ GAG chains is provided by the sulfation pattern of HS that significantly influences HSPGs’ interactions with their ligands [19,20,21,22,23,24,25]. According to current understanding, the sulfation pattern of HSPGs expressed by a given cell is the same, regardless of the core protein [19,26,27,28,29]. Altered HSPG expression is associated with several pathological conditions, including Alzheimer’s disease (AD), wherein the increased expression of HSPGs, including the neuronal SDC (i.e., SDC3), correlates with neuronal vulnerability to disease pathology [30,31].

SDCs are the only transmembrane HSPG family [13,14,16,19,32,33]. SDCs exhibit tissue-specific expression: SDC1 is expressed by epithelial and plasma cells, SDC2 by endothelial cells and fibroblasts, while SDC3 is predominant in neurons. SDC4 is a ubiquitously expressed isoform of the SDC family [14,16,19,32,34,35]. SDCs share a similar structure: a short cytoplasmic domain, a conserved, single-span transmembrane domain (TM), and an extracellular domain with attachment sites for three to five HS or chondroitin sulfate (CS) chains [16]. The HS chains of SDCs enable interactions with many extracellular ligands, while their cytoplasmic domains facilitate intracellular signaling cascades [14,16,19,32,36]. Attachment to SDCs triggers the oligomerization of the bound ligands, arising from the multivalent ligand-binding sites of SDCs’ GAG chains and the core protein-mediated oligomerization of SDCs themselves [16,19,37,38]. Besides its GAG chains, the neuron-predominant SDC3 also possesses several mucin-rich regions, while the extracellular domain of SDC4 also comprises a cell-binding domain (CBD) mediating cell to cell attachment [16,19,33,39].

Among SDCs, SDC1 has already been reported to enhance the cellular uptake of ApoE containing lipoproteins, thus facilitating hepatic lipoprotein clearance [40]. Besides the liver, the CNS shows the highest ApoE expression. ApoE is also associated with AD in an isoform-dependent manner in the brain: ApoE4 increases the AD risk 4- to 14-fold, while ApoE2 decreases it [4,6,9,41]. It has also been shown that ApoE is co-deposited with amyloid-beta (Aβ) in amyloid plaques [9,42]. Thus ApoE has been suggested to be involved in plaque formation by directly interacting with Aβ or affecting Aβ clearance [43,44]. ApoE isoforms interact differently with Aβ: ApoE4 promotes plaque formation via a stronger association with Aβ than ApoE3 and ApoE2 [43,45].

Previously, we demonstrated that SDCs, particularly the neuronal SDC3, enhances amyloid pathology by facilitating the aggregation of Aβ and increasing Aβ internalization, especially when Aβ is already present in the aggregated form [16]. Utilizing our previously established SDC-specific cell lines and assays, we also explored the interplay of ApoEs with SDCs in influencing Aβ uptake and fibrillation, two critical steps in plaque formation. Our results show the isoform-specific nature of ApoEs in the interaction with SDCs and reveal further details of the intricate effect of ApoEs on amyloid pathology.

## 2. Results

### 2.1. SDCs Increase the Cellular Internalization of ApoE Isoforms

**Figure 1:**
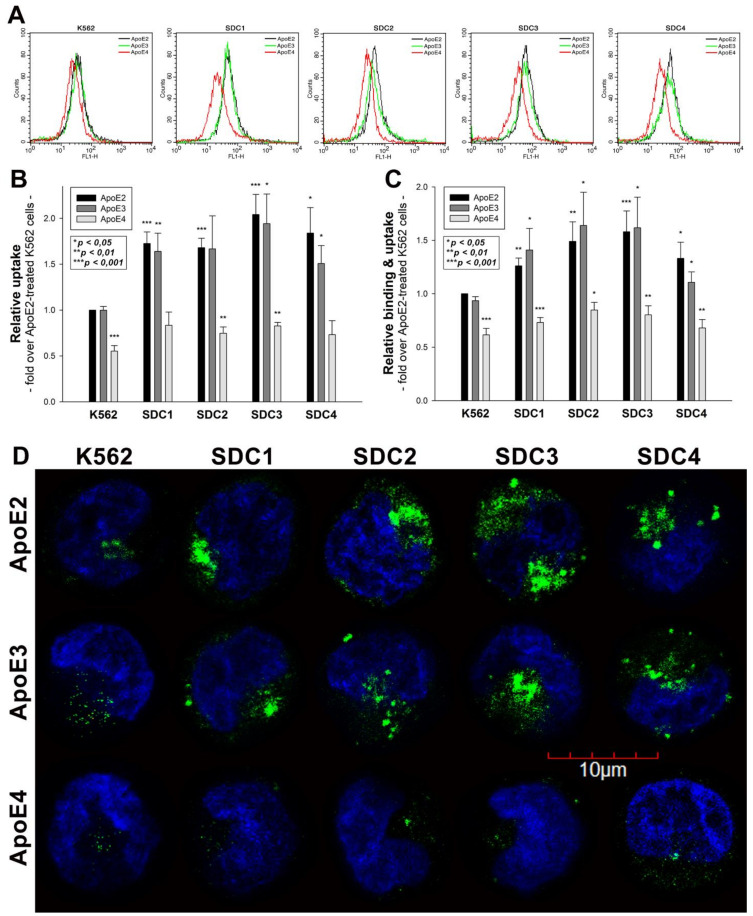
Cellular uptake of ApoEs into WT K562 cells and SDC transfectants. WT K562 cells and SDC transfectants were treated with either of the FITC-labeled ApoE isoforms for 3 h at 37 °C. Cellular uptake of ApoE isoforms was then measured with flow cytometry and confocal microscopy. (**A**) Flow cytometry histograms representing intracellular fluorescence WT K562 cells and SDC transfectants treated with FITC-labeled ApoEs. (**B**,**C**) Detected intracellular and cellular fluorescence intensities were normalized to FITC-ApoE2-treated WT K562 cells as standards. The bars represent the mean ± SEM of six independent experiments. Statistical significance vs. standards (i.e., FITC-ApoE2-treated WT K562 cells) was assessed with analysis of variance (ANOVA). * *p* < 0.05; ** *p* < 0.01; *** *p* < 0.001. (**D**) Confocal microscopic visualization of ApoE uptake into K562 cells and SDC transfectants. The nuclei of cells were stained with DAPI. Representative images of three independent experiments are shown. Scale bar = 10 μm.

The cellular uptake of ApoE isoforms was investigated with SDC-specific cellular models [14,16,32]. Human myelogenous leukemia K562 cells, a cell line with shallow levels of endogenous HSPG and no caveolae expression, reportedly express no SDCs or GPCs [14,16,19,32,46,47,48]. K562 cells’ poor HSPG expression and their inability to form caveolae, the source of caveolar endocytosis, renders K562 cells an ideal cell line to study the effect of SDCs overexpression on ApoE uptake while avoiding the disturbing effects of other HSPGs or caveolae-mediated endocytosis [16,19]. Thus, SDC isoforms overexpressing stable transfectants were created in K562 cells (Supplementary Figure S1A) [14,16,19,32,33]. As the role of HS chains in ApoE attachment has already been evaluated [8,10,11,12], SDC-specific stable transfectants were standardized according to HS expression (Supplementary Figure S1B,C). Hence SDC transfectants with almost equal HS expression were chosen, and along with WT K562 cells, treated with FITC-labeled ApoE isoforms (ApoE2, 3 and 4). (It has to be noted that SDC overexpression left LRP1 expression unaffected. Thus, the LRP1 expression of SDC transfectants was the same as in WT K562 cells (Supplementary Figure S2A,B)). After 3 h of incubation with FITC-labeled ApoEs, the cells were washed, and ApoE uptake was quantified with flow cytometry. The internalization of ApoE isoforms was measured by adding trypan blue (dissolved at a concentration of 0.25% in ice-cold 0.1 M citrate buffer) 1 min before the flow cytometric analyses, hence the fluorescence of extracellularly attached ApoE isoforms was quenched [14,16]. Flow cytometry studies of ApoE-treated SDC transfectants showed that SDC overexpression increased the cellular uptake of ApoEs in an isoform-dependent manner: among SDCs, neuronal SDC3 increased ApoE internalization the best (Figure 1A,B). Among ApoEs, SDCs increased ApoE2 and ApoE3 uptake most effectively, while SDCs’ effect on increasing ApoE4 uptake was lower (Figure 1A,B). Compared to ApoE-treated WT K562 cells, the overall cellular fluorescence of FITC-ApoE-treated SDC transfectants also increased, indicating that SDC overexpression increased both the cellular binding and the uptake of ApoEs (Figure 1C). (Compared to untreated control cells, ApoE treatment did not affect cell viability as measured with flow cytometry. As cell viability remained constant, it could not influence the detected difference in fluorescence intensities of ApoE-treated cells (Supplementary Figure S3)). In line with the flow cytometry measurement, confocal microscopy studies also revealed the more intense intracellular fluorescence of FITC-ApoE2- and ApoE3-treated SDC transfectants, while, compared to the other ApoE isoforms, the uptake of ApoE4 remained relatively modest (Figure 1D).

**Figure 2:**
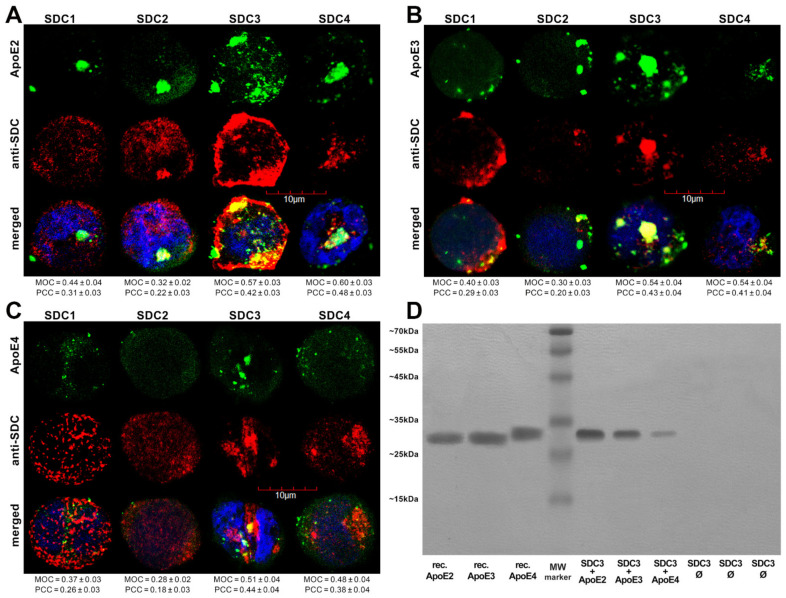
Colocalization of ApoEs and SDCs. SDC transfectants were treated with either of the FITC-ApoEs for 3 h at 37 °C. After incubation, the cells were permeabilized and treated with the respective APC-labeled SDC antibody. Nuclei of cells were stained with DAPI and colocalization was then analyzed with confocal microscopy. (**A–C**) Confocal microscopic images of SDC transfectants treated either of the FITC-ApoEs and the respective APC-labeled SDC antibody. Representative images of three independent experiments are shown. Scale bar = 10 μm. MOC or PCC ± SEM for the colocalization of SDCs with FITC-ApoEs was calculated by analyzing 18 images with ~7 cells in each image (from three separate samples). (**D**) SDS-PAGE showing FITC-labeled ApoEs immunoprecipitated with SDC3 from extracts of SDC3 transfectants. Lanes 1–3: 0.5 µg of FITC-ApoEs. Lane 4: molecular weight (MW) marker. Lanes 5–7: immunoprecipitates of SDC3 transfectants treated with either of the FITC-ApoEs. Lanes 8-10: immunoprecipitates of untreated SDCs transfectants. Standard protein size markers are indicated on the left. Image acquisition was carried out with UVITEC Alliance Q9 Advanced Imager.

Utilizing the Mander’s overlap coefficient (MOC, showing the percentage of pixels overlapped on a scale of 0 to 1 [49,50]) to measure the degree of overlap between ApoEs and SDCs, colocalization studies revealed that about half of the ApoEs overlap with either SDC3 or SDC4 (i.e., MOCs ~0.50 as shown in Figure 2A–C). At the same time, the MOCs of SDC1 with ApoEs were slightly lower (MOCs ~0.40, see Figure 2A–C). According to the detected MOC values, the overlap of all ApoEs with SDC2 was the lowest. To reveal a more detailed picture of colocalization, we also analyzed the correlation of ApoEs and SDCs with the Pearson correlation coefficient (PCC) [51]. The detected PCC values showed a moderate correlation (i.e., PCC ~0.4) of ApoEs with SDC3 and 4 (Figure 2A–C), while the PCC values for ApoEs with SDC1 were slightly lower (i.e., PCC ~0.3). Similar to MOC, the PCCs of ApoEs with SDC2 were the lowest, suggesting a small correlation (Figure 2A–C). When immunoprecipitated with SDC3 from extracts of ApoE-treated SDC3 transfectants, ApoE2 exhibited the strongest band, followed by ApoE3 and 4 (Figure 2D and Supplementary Figure S4A), thus confirming the previously detected differences in the internalization efficacy of the ApoE isoforms (ApoE2 ≥ ApoE3 > ApoE4, as shown in Figure 1).

### 2.2. The Colocalization of SDC3 with ApoE in SH-SY5Y Cells

**Figure 3:**
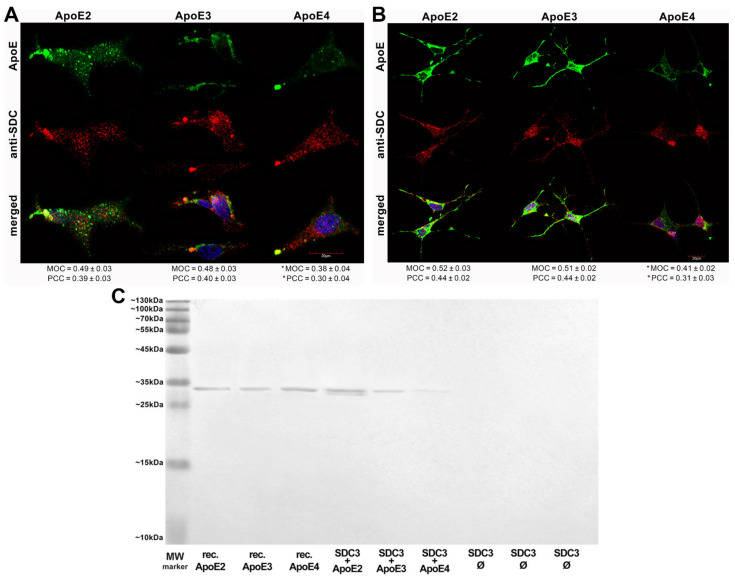
Colocalization of ApoEs with SDC3 in SH-SY5Y cells. (**A**,**B**) Undifferentiated (**A**) or differentiated (**B**) SH-SY5Y cells were treated with either of the FITC-ApoEs for 3 h at 37 °C. After incubation, the cells were permeabilized and treated with the APC-labeled SDC3 antibody. Nuclei of cells were stained with DAPI and colocalization was then analyzed with confocal microscopy. Representative images of three independent experiments are shown. Scale bar = 20 μm. MOCand PCC ± SEM, for the colocalization of SDCs with FITC-ApoEs, was calculated by analyzing 21 images with ~7 cells in each image (from three separate samples). (**C**) SDS-PAGE showing FITC-labeled ApoEs immunoprecipitated with SDC3 from extracts of differentiated SH-SY5Y. Lane 1: MW marker. Lanes 2–4: 0.5 µg of FITC-ApoE2,3 and 4, respectively. Lanes 5–7: immunoprecipitate of SH-SY5Y cells treated with either of the FITC-ApoEs. Lanes 8–10: immunoprecipitate of untreated SH-SY5Y cells. Standard protein size markers are indicated on the left. Image acquisition was carried out with UVITEC Alliance Q9 Advanced Imager.

After assessing the contribution of SDCs to the cellular uptake of ApoE isoforms in a cellular model with a fairly low HSPG expression, we advanced to studies on SH-SY5Y cells, a widely used in vitro model of neuronal cell lines [52,53,54]. Compared to K562 cells, the HS expression of wild-type SH-SY5Y cells is more pronounced, while their SDC3 expression is still very modest [16]. Microscopic colocalization studies on undifferentiated and differentiated SH-SY5Y cells gave MOC values of ~0.5 for SDC3 with ApoE2 or ApoE3 (Figure 3A,B), demonstrating that about half of ApoE2 and 3 colocalizes with SDC3 during internalization. The MOC value for SDC3 and ApoE4 was slightly lower (Figure 3A,B). Quantifying colocalization with PCC revealed values ~0.4 for ApoE2/3 and SDC3 and ~0.3 for ApoE4 and SDC3, suggesting a moderate colocalization. Following immunoprecipitation with SDC3 from extracts of ApoE-treated differentiated SH-SY5Y cells, ApoE2 exhibited the strongest band, followed by ApoE3 and 4 (Figure 3C and Supplementary Figure S4B), further confirming the previously revealed differences in the internalization efficacy of the ApoE isoforms.

### 2.3. Overexpression of SDC3 Increases ApoE Uptake in Neurons

**Figure 4:**
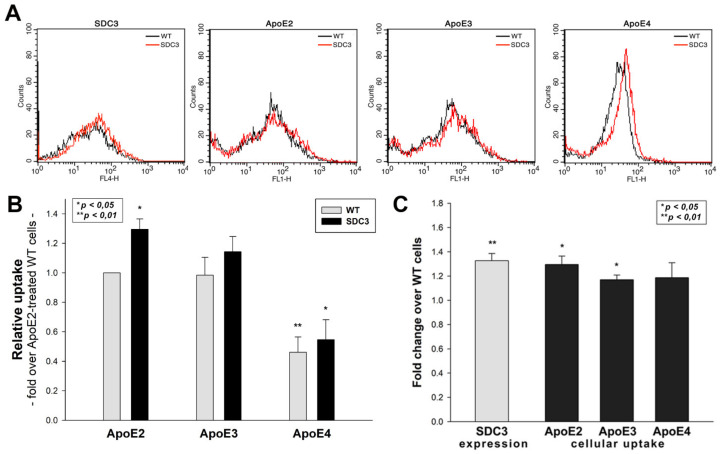
SDC3 overexpression increases ApoE uptake into SH-SY5Y cells. SDC3-overexpressing clones created in differentiated SH-SY5Y cells were selected by measuring SDC3 expression with flow cytometry. (**A**) Flow cytometry histograms representing SDC3 expression levels and intracellular fluorescence of FITC-ApoE-treated differentiated WT SH-SY5Y cells and SDC3 transfectants, created in differentiated SH-SY5Y cells. SDC3 transfectants and WT SH-SY5Y cells were treated with FITC-labeled ApoEs at 37 °C for 3 h and then processed for uptake studies. (**B**) Detected intracellular fluorescence intensities were normalized to FITC-ApoE2-treated WT SH-SY5Y cells as standards. The bars represent the mean ± SEM of three independent experiments. Statistical significance vs. standards was assessed with ANOVA. (**C**) Fold change in SDC3 expression, along with ApoE uptake following SDC3 overexpression in differentiated SH-SY5Y cells. The bars represent the mean ± SEM of three independent experiments. The statistical significance vs. ApoE2-treated WT SH-SY5Y cells as standards was assessed with ANOVA. * *p* < 0.05; ** *p* < 0.01.

Overexpressing SDC3 in differentiated SH-SY5Y cells increased uptake for all ApoE isoforms (Figure 4A–C). Among ApoEs, ApoE2 exhibited the highest internalization efficacy, while ApoE4 the lowest (Figure 4C). The extent of increase in ApoE uptake was similar to the increase in SDC3 expression. (It also has to be noted that SDC3 overexpression left the LRP1 expression of SH-SY5Y cells unaffected (Supplementary Figure S5A,B). Cellular viability was also measured during the uptake studies: the ApoE treatment of both WT and SDC3-overexpressing SH-SY5Y cells left cellular viability unaffected (Supplementary Figure S6).

### 2.4. ApoEs Modulate Amyloid Pathology by Interfering with Aβ1–42 Fibrillation and Iptake

**Figure 5:**
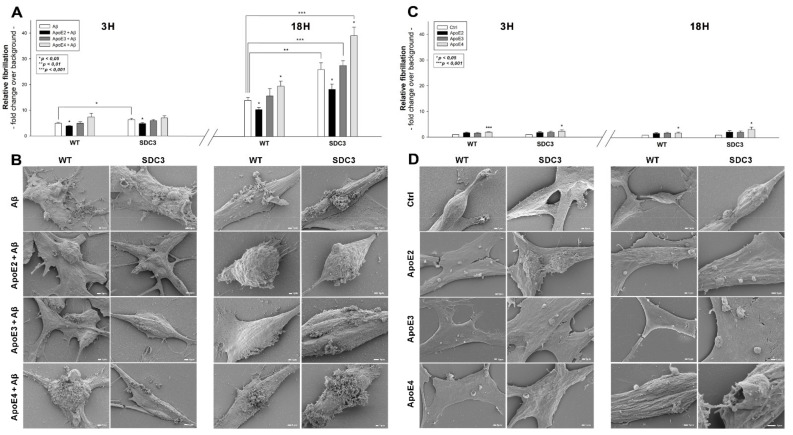
The effect of ApoEs on Aβ1-42 fibrillation in WT and SDC3-overexpressing differentiated SH-SY5Y cells. WT and SDC3-overexpressing differentiated SH-SY5Y cells were incubated with either of the ApoE isoforms at 37 °C. Thirty minutes later, some of the cells were treated with Aβ1–42 and incubated for either 3 h or 18 h (**A**,**B**). After incubation, fibrillation was analyzed with either ThT staining or electron microscopy. (**A,C**) Results of ThT fluorescence assays after 3 h and 18 h of incubation with Aβ1–42 in the presence or absence of ApoEs (**A**) or the ApoE isoforms alone (**C**). Amyloid fluorescence is expressed as fold change over background ThT fluorescence. The bars represent the mean ± SEM of four independent experiments. In the case of Aβ1–42 treatment (**A**), statistical significance was assessed vs. Aβ1–42-only treated cells with ANOVA. For cells receiving ApoE treatment only (**B**), statistical significance was assessed vs. untreated controls. * *p* < 0.05; ** *p* < 0.01; *** *p* < 0.001. (**B,D**) Scanning electron microscope visualization of WT and SDC3-overexpressing differentiated SH-SY5Y cells at 3 h and 18 h of incubation with Aβ1–42 in the presence or absence of ApoEs (**B**) or the ApoE isoforms alone (**D**). Representative images of three independent experiments are shown. Scale bar = 1 μm.

Previously, we have shown that SDCs, especially the neuronal SDC3, significantly influence amyloid pathology by modulating the cellular internalization and fibrillation of Aβ1–42 [16]. Utilizing SDC3-overexpressing differentiated SH-SY5Y cells, we explored the effect of ApoEs on SDC3-induced changes in Aβ1–42 aggregation. Thioflavin T (ThT) fluorescence studies revealed the slight fibrillation of Aβ1–42 at 3 h and a more intense one at 18 h, based on Figure 5A,B. SDC3 overexpression increased the fibrillation of Aβ1–42 at 18 h, while its effect at 3 h of incubation was insignificant (Figure 5A). ApoEs exerted differential effects on Aβ1–42 aggregation: ApoE2 decreased Aβ1–42 aggregation, even in SDC3-overexpressing transfectants at 18 h, while ApoE4 markedly increased it, both in the case of the WT SH-SY5Y cells and the SDC3 transfectants. Scanning electron microscopy further confirmed the effect of ApoEs on Aβ1–42 aggregation: the representative images of Figure 5B clearly show that ApoE2 reduced, and ApoE4 increased, Aβ1–42 aggregation. These effects of ApoE2 and 4 were rather striking on SDC3-overexpressing SH-SY5Y cells, where ApoE2 reduced the number of aggregates, while ApoE4 further increased them.

Recent studies have explored small regions with amyloidogenic properties in the amino acid sequences of apoE3 and 4, showing that these aggregation hot spots would drive the self-assembly of amyloid-like fibrils [55]. Thus, we also examined the fibrillation of ApoEs isoforms in the absence of Aβ1–42. ThT fluorescence studies on ApoE-treated SH-SY5Y cells and SDC3 transfectants revealed the fibrillation of ApoE4 at both 3 and 18 h of incubation (along with the fibrillation of ApoE3 at 3 h, Figure 5C). Compared to Aβ1–42 (Figure 5C), the extent of fibrillation of ApoE4 was less profound, as shown by the lower ThT fluorescence values obtained on ApoE-treated cells. Electron microscopic studies also show the formation of fibrillar-like aggregates on the surfaces of ApoE4-treated cells, especially at 18 h of incubation. The presence of such small fibrils on ApoE3-treated cells was less evident, while electron microscopy did not reveal ApoE2 fibrils on ApoE2-treated SH-SY5Y cells and SDC3 transfectants (Figure 5D).

### 2.5. ApoEs Modulate Aβ1–42 Uptake

**Figure 6:**
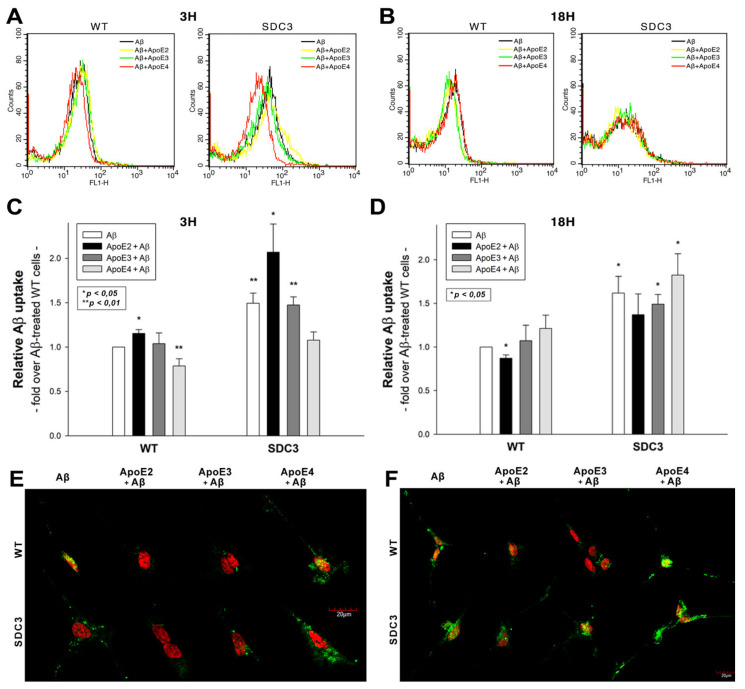
The effects of ApoEs on Aβ1–42 uptake in WT and SDC3-overexpressing differentiated SH-SY5Y cells. WT and SDC3-overexpressing differentiated SH-SY5Y cells were preincubated with different ApoEs for 30 min at 37 °C. The cells were then treated with FITC-labeled or, in case of fibrillation studies, unlabeled Aβ1–42 for either 3 or 18 h. After incubation, the cellular uptake of Aβ1–42 was analyzed. (**A,B**) Flow cytometry histograms representing the intracellular fluorescence of differentiated WT and SDC3-overexpressing differentiated SH-SY5Y cells treated with FITC-labeled Aβ1–42 for 3 (**A**) and 18 h (**B**) in the absence or presence of ApoEs. (**C,D**) Detected intracellular fluorescence intensities were normalized to FITC-Aβ1–42-only treated WT SH-SY5Y cells as standards. The bars represent the mean ± SEM of four independent experiments. Statistical significance vs. standards was assessed with ANOVA. * *p* < 0.05; ** *p* < 0.01. (**E,F**) Confocal microscopic visualization of ThT-labeled Aβ1–42 fibrils in WT and SDC3-overexpressing differentiated SH-SY5Y cells at 3 h (**E**) and 18 h (**F**) of incubation. The nuclei of cells were stained with DRAQ5. Representative images of three independent experiments are shown. Scale bar = 20 μm.

Utilizing WT and SDC3-overexpressing differentiated SH-SY5Y cells, we also explored the effect of ApoEs on Aβ1–42 uptake. In line with our previous findings [16], SDC3 overexpression increased the cellular uptake of Aβ1–42 at both 3 and 18 h of incubation (Figure 6A–D). ApoEs exerted various effects on Aβ1–42 uptake: at 3 h ApoE2 increased Aβ1–42 internalization, while ApoE4 reduced it (Figure 6A,C). This effect of ApoEs on Aβ1–42 uptake was reversed at 18 h of incubation: ApoE4 increased Aβ1–42 uptake, while ApoE2 and 3 reduced it (Figure 6B,D). (Compared to untreated control cells, ApoE treatment did not affect cell viability when measured with flow cytometry. As cell viability remained constant, it could not influence the detected difference in fluorescence intensities of ApoE-treated cells (Supplementary Figure S7)). Confocal microscopy also showed an increased presence of ThT-labeled aggregates at 18 h in WT and SDC3-overexpressing differentiated SH-SY5Y cells, which was reduced in the case of samples pretreated with ApoE2. ApoE4, on the other hand, increased the number of ThT-labeled aggregates, indicating the increased presence of Aβ1–42 aggregates (Figure 6E,F).

### 2.6. Colocalization of ApoE4 with Aβ1–42 Fibrils

**Figure 7:**
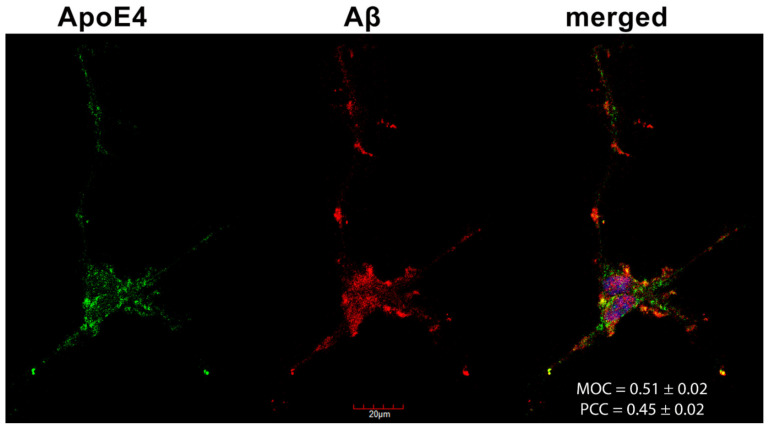
The colocalization of ApoE4 and Aβ1–42 fibrils. Differentiated SH-SY5Y cells were preincubated with FITC-ApoE4 and then treated with Aβ1–42 for 18 h. After incubation, Aβ1–42 fibrils were stained with Congo Red (CR), while the nuclei of cells were stained with DAPI. The colocalization of ApoE4 with CR-stained Aβ1–42 fibrils was then analyzed with confocal microscopy. Representative images of three independent experiments are shown. Scale bar = 20 μm. MOC and PCC ± SEM, for the colocalization of ApoE4 with CR-stained Aβ1–42 fibrils, was calculated by analyzing 21 images with ~5 cells in each image (from three separate samples). Scale bar = 20 μm.

Confocal microscopy studies revealed that about half of the Congo Red-stained Aβ1–42 fibrils and ApoE4s overlap (i.e., MOC = 0.51 and PCC = 0.45, as shown in Figure 7), confirming previous findings on the colocalization of amyloid plaques and ApoE4 [44,56,57].

## 3. Discussion

Humans possess three major alleles of the APOE gene [58]. The three human ApoE isoforms, namely, ApoE2, ApoE3, and ApoE4, differ by single amino acid substitutions at positions 112 and 158, ApoE2 (Cys-112, Cys-158), ApoE3 (Cys-112, Arg-158), and ApoE4 (Arg-112, Arg-158) [59,60]. ApoE4 is the most prevalent genetic risk factor of AD, while ApoE2 decreases AD risk [4,6,59,60,61,62]. HSPGs are among the major cellular receptors for ApoEs. Neuronal HSPGs, on the other hand, promote AD and amyloid pathology by modulating brain Aβ clearance and aggregation [31]. Among the cell membrane HSPGs, the expression of SDC3 and SDC4 is significantly elevated in human AD brains [31].

In one of our previous papers, we demonstrated how SDCs, especially the neuronal SDC3, trigger amyloid pathology by influencing the cellular uptake and fibrillation of Aβ1–42 [16]. Interestingly, this effect of SDCs was independent of the cell type, and HS side chains were only partially responsible for the SDC-mediated effects on Aβ1–42. On the other hand, HS chains are viewed as the primary binding site for ApoEs on HSPGs. Thus the interaction of ApoEs with heparin has been intensely studied. Although initial SPR studies reported the similar affinities of the three ApoE isoforms for heparin [63], later biochemical analyses indicated that ApoE2 tends to bind to heparin with somewhat less affinity than ApoE3 and ApoE4 [64].

Considering the significance of the scientific evidence on the effect of the interaction of ApoEs with HSPGs in influencing the pathomechanism of AD, we decided to explore the interplay of ApoEs with SDCs in key cellular events of amyloid pathology. Utilizing SDC-specific assays created in a cell line (K562) devoid of SDCs and other major HSPGs enabled us to perform the molecularly focused analysis of SDCs’ interaction with ApoEs. As the HS sulfation pattern is the same for every HS chain that a given cell type synthesizes, investigating SDCs’ interactions with ApoEs in SDC transfectants created in a given cell type (i.e., K562 cells) helped us to study how isoform-specific effects of SDCs (i.e., those beyond the HS chains) would influence the interactions with ApoEs. Standardizing the created SDC transfectants according to HS content also helped to explore the HS-independent effects of SDCs on ApoEs. Cellular uptake studies with these SDC-overexpressing transfectants showed the increased cellular uptake of all ApoEs due to SDC overexpression. Among ApoEs, ApoE2, the isoform highly defective in LDLR binding activity (<2% of normal ApoE3 activity) and possessing significant binding activity to LRP (40–50% of ApoE3) and HSPG (50–90% of ApoE3) [11,64], was internalized the best, especially by SDC3 transfectants. On the other hand, ApoE4, the isoform with a high affinity towards heparin and HS [64], exhibited weak internalization compared to ApoE2 or ApoE3. Thus, the superior internalization of ApoE2 into SDC-overexpressing cells must be attributed to the HS-independent effects of SDCs. Having explored the SDC-mediated increase in the cellular uptake of ApoEs, along with the superior internalization of ApoE2 being influenced by SDC-specific effects irrespective of the HS chains, we also assessed how the interaction of ApoEs with SDCs contributes to the previously revealed effect of SDC3 on Aβ1–42 uptake and aggregation. Uptake studies showed that ApoEs exert opposite effects on Aβ1–42 uptake: ApoE2 increased the cellular uptake of monomeric Aβ1–42, while decreasing it once Aβ1–42 is aggregated. Thus, by increasing monomeric Aβ1–42 uptake, ApoE2 prevents the extracellular accumulation and following fibrillation of Aβ1–42. ApoE2 continued to facilitate the uptake of monomeric Aβ1–42 in SDC3 transfectants, suggesting that the increased monomeric Aβ1–42 internalization activity due to SDC3 overexpression might be a protective activity of neurons to prevent the neuronal buildup of Aβ plaques. ApoE4, on the other hand, acted contrarily to ApoE2: ApoE4 reduced the uptake of monomeric Aβ1–42, thus facilitating the buildup and subsequent aggregation of Aβ1–42. The previous finding that ApoE4 stabilizes Aβ oligomers [57,65] was confirmed with confocal microscopic studies showing that 50% of Aβ1–42 fibrils colocalize with ApoE4. Fibrillation studies also revealed ApoE4′s propensity to form fibrillar aggregates; however, the size of ApoE4-based fibrils was markedly smaller than those of Aβ1–42. Regardless, these studies further confirmed that due its the intrinsic aggregation-prone regions, ApoE4 (and to a lesser extent, ApoE3) self-assembles into amyloid fibrils in vitro, further triggering (and stabilizing) the fibrillation of Aβ1–42 [55]. Utilizing these very basic SDC-focused cellular assays not only helped to reveal the intricate effect ApoEs exert on Aβ1–42, but also expanded our knowledge of the SDC3-mediated effects of amyloid pathology.

Overall, our data confirm previous studies on the interaction of ApoEs with HSPGs, along with the effects of ApoEs on Aβ1–42 uptake and aggregation. On the other hand, our studies also provide new insight into the molecular interaction of ApoEs with SDCs, a transmembrane proteoglycan family with emerging significance in the pathomechanism of neurodegeneration. Studies carried out on SDC-specific models with standardized HS content suggest that ApoE–SDC interaction goes beyond the HS chains. The intricate effects of ApoEs on SDC-mediated Aβ1–42 uptake and fibrillation also reveal novel insights into the sophisticated and opposing effects ApoEs have on amyloid pathology. Utilizing SDC-specific cellular models has uncovered molecular details of ApoE cellular biology, and deepens our molecular understanding of the ApoE-dependent mechanisms of amyloid disorders.

## 4. Materials and Methods

### 4.1. Peptides, Proteins and Fluorescent Labeling

Aβ1–42 (i.e., Aβ1–42 trifluoroacetate salt) and its FITC-labeled derivative (FITC-εAhx-Aβ1–42) trifluoroacetate salt was purchased from BACHEM (Bubendorf, Switzerland) (cat. no. H-8146 and H-7666) while recombinant ApoEs were purchased from Peprotech Germany, Hamburg, Deutschland. Aβ1–42 peptides (either unlabeled or fluorescently labeled) were kept lyophilized, while fresh Aβ1–42 solutions were prepared by dissolving the lyophilized Aβ1–42 in DMSO immediately prior to the experiments. Fluorescent labeling of ApoE isoforms was performed with the Pierce™ FITC Labeling Kit according to the manufacturer’s instructions (Thermo Fisher Scientific, Waltham, MA, USA, cat. no. 53027). The calculated yield of the labeling was 4.5 mol dye/ApoE. The even fluorescent labeling of the ApoE isoform was confirmed with spectrophotometry (Metertech UV/VIS) and gel electrophoresis (Supplementary Figure S8).

### 4.2. SDC Constructs, Cell Culture and Transfection

Full-length SDC1-4 transfectants, established in K562 cells (ATCC CCL-243), were created as described previously [14,16,19,33]. Stable SDC transfectants were selected by measuring SDC expression with flow cytometry (Becton Dickinson FACScan, Franklin Lakes, NJ, USA) using APC-labeled SDC antibodies specific to the respective SDC isoform (all RnD Systems, Minneapolis, MN, USA; SDC1: monoclonal rat IgG1 Clone #359103, cat. no. FAB2780A [66,67,68]; SDC2: monoclonal rat IgG2B Clone #305515, cat. no. FAB2965A [69,70,71]; SDC3: polyclonal goat IgG, cat. no. FAB3539A [69,72]; SDC4: monoclonal rat IgG2a clone #336304, cat. no. FAB29181A [16,19,33]). In the case of SDC3-overexpressing SH-SY5Y cells, the transfection and selection of SDC3-overexpressing clones was carried out as described previously [19]. SDC3-overexpressing SH-SY5Y clones, along with WT SH-SY5Y cells, were then grown in Advanced MEM medium (Thermo Fischer Scientific, Waltham, MA, USA) supplemented with 10% FCS (Gibco, New York, NY, USA) at 37 °C in a humified 5% CO2 air environment. The LRP1 expression of WT K562 and SH-SY5Y cells along with SDC transfectants was measured with flow cytometry using FITC-labeled LRP1 antibodies (Santa Cruz Biotechnology, Dallas, Texas, USA, cat.no. sc-19616 FITC).

### 4.3. Differentiation Protocol of SH-SY5Y Cells

The differentiation of SH-SY5Y cells was carried out as described previously [16,19], before treating the cells with FITC-labeled ApoEs and FITC Aβ1–42, purchased from BACHEM (Bubendorf, Switzerland) (cat. no. H-8146 and H-7666).

### 4.4. Flow Cytometry Analysis of HS and SDC

As HS was shown to attach to ApoE, the HS expression of wild-type (WT) K562 cells, SDC transfectants and SH-SY5Y cells was measured with flow cytometry using the anti-human HS antibody (10E4 epitope (Amsbio, Abingdon, UK)) and FITC- or Alexa 647-labeled goat anti-mouse IgG (Sigma, St. Louis, MO, USA), as described previously [16,19,33]. SDC transfectants with an almost equal amount of HS expression were selected for further uptake studies.

### 4.5. Flow Cytometry Analysis of Cellular Uptake

WT K562 and SH-SY5Y cells, SDC transfectants and differentiated SH-SY5Y cells were utilized to quantify the internalization of fluorescently labeled Aβ1–42 or ApoEs. Briefly, 6 × 10^5^ cells/mL in DMEM/F12 medium (with 10% FCS) were incubated with FITC-labeled Aβ1–42 or ApoEs (at a concentration of 5 μM and 500 nM, respectively) for various amounts of time (3 and 18 h) at 37 °C. The effect of ApoEs on Aβ1–42 uptake was measured by preincubating the cells with either of the unlabeled ApoE isoforms for 30 min at 37 °C at a concentration of 500 nM, before treating the cells with 5 μM of FITC-labeled Aβ1–42. After incubation, the cells were washed twice in ice-cold PBS and progressed towards flow cytometry. Then the cells (WT K562, SH-SY5Y and SDC transfectants) were resuspended in 0.5 mL of physiological saline. Equal volumes of this suspension and a stock solution of trypan blue (Merck KGaA, Darmstadt, Germany; 500 μg/mL dissolved in ice-cold 0.1 M citrate buffer at pH 4.0) were allowed to mix for 1 min before the flow cytometric analyses. In this way, the sample’s pH was lowered to pH 4.0, thereby optimizing the quenching effect of trypan blue to quench the extracellular fluorescence of surface-bound fluorescent proteins [14,16]. Cellular uptake and attachment was then measured by flow cytometry using an FACScan (Becton Dickinson, Franklin Lakes, New Jersey, USA). Cellular attachment was calculated by subtracting intracellular fluorescence (i.e., those quenched with trypan blue or trypsin) from the measures of overall fluorescence. A minimum of 10,000 events per sample was analyzed. The viability of cells was determined by appropriate gating in a forward scatter against side scatter plot to exclude dead cells and debris [14,16].

### 4.6. Microscopic Visualization of Cellular Uptake

Internalization of the fluorescently labeled (FITC) Aβ1–42 or ApoEs was visualized by confocal laser scanning microscopy. WT SH-SY5Y and WT K562 cells, along with SDC transfectants and SDC3-overexpressed SH-SY5Y cells, were grown on poly-D-lysine-coated glass-bottom 35 mm culture dishes (MatTek Corp. Ashland, MA, USA). After 24 h, the cells were preincubated in DMEM/F12 medium (supplemented with 10% FCS) at 37 °C for 30 min before incubation with the FITC-labeled Aβ1–42 or ApoEs at a concentration of 5 μM and 500 nM, respectively. After incubation, the cells were rinsed two times with ice-cold PBS, fixed in 4% paraformaldehyde (Sigma, St. Louis, MO, USA), and the nuclei were stained with DAPI (1:5000, Sigma, St. Louis, MO, USA) for 5 min [16,19]. For the colocalization studies, after fixation, the cell membranes were permeabilized (1% Triton X-100), followed by 1 h of treatment with APC-labeled SDC antibodies (5:100). The samples were then rinsed three times with PBS containing 1% goat serum and 0.1% Triton X-100, then stained with DAPI (1:5000) for 5 min, washed three times with PBS, and embedded in Fluoromount G (SouthernBiotech, Birmingham, AL, USA). The distribution of fluorescence was then analyzed on an Olympus FV1000 confocal laser scanning microscope as described previously [14,16,19]. The photomultiplier gain and laser power were identical within each experiment. The FV10-ASW software was used for image acquisition by confocal microscopy. For visualizing the internalization of oligomeric Aβ1–42, WT K562 cells and SDC transfectants grown on poly-D-lysine-coated glass-bottom 35 mm culture dishes were incubated with Aβ1–42 (BACHEM, Bubendorf, Switzerland) at a concentration of 5 µM (in DMEM/F-12 without Phenol Red) at 37 °C for 18 h, then treated with Thioflavin T (ThT, Sigma, St. Louis, MO, USA) at a concentration of 25 µM, or Congo Red stain 1%, for 10 min at 37 °C and rinsed two times with ice-cold PBS. After fixation in 4% paraformaldehyde (Sigma, St. Louis, MO, USA), the nuclei were stained with either DRAQ5 or DAPI (1:5000) for 5 min, then after three washes with PBS, the samples were embedded in Fluoromount G and the distribution of fluorescence was analyzed on an Olympus FV1000 confocal laser scanner, as described above. For colocalization analyses, the images were analyzed in the ImageJ software (NIH, Bethesda, Maryland, US) with the plug-in JACoP, as described previously [16,19,33]. In total, 21 images (7 images per sample, experiment performed in triplicate) were analyzed, and the data are presented as mean ± SEM [16,19,33].

### 4.7. Co-Immunoprecipitation Experiments

Stable SDC3 transfectants created in K562 or differentiated WT SH-SY5Y cells were incubated with or without FITC-labeled ApoEs at a concentration of 500 nM for 18 h at 37 °C. After incubation, the cells were washed twice with ice-cold PBS and treated with cold Pierce IP lysis buffer. Then the cells were scraped off into clean Eppendorf tubes, put on a low-speed rotating shaker for 15 min and centrifuged at 14,000× *g* for 15 min at 4 °C. The supernatant was then transferred to new tubes and combined with 5 µg of the human SDC3 antibody (R&D Systems, Minneapolis, MN, USA). The antigen sample/SDC3 antibody mixture was then incubated overnight at 4 °C with mixing. The antigen sample/SDC3 antibody mixture then was added to a 1.5 mL microcentrifuge tube containing pre-washed Pierce Protein A/G Magnetic Beads (Thermo Fisher Scientific, Waltham, MA, USA). After incubation at room temperature for 1 h with mixing, the beads were collected with a MagJET Separation Rack magnetic stand (Thermo Fisher Scientific, Waltham, MA, USA), and supernatants were discarded. To eluate the antigen, 100 µL of SDS-PAGE reducing sample buffer was then added to the tubes and samples were heated at 96 °C for 10 min, and the samples proceeded to SDS-PAGE. Image acquisition was carried out with a UVITEC Alliance Q9 Advanced Imager (Uvitec Ltd., Cambridge, UK) [16,19,33]. The intensity of the bands was analyzed with the NineAlliance© software.

### 4.8. Thioflavin T Binding Assays

Differentiated WT and SDC3-overexpressing SH-SY5Y cells were seeded into black-sided, clear-bottom 96-well microplates (Corning, New York, NY, USA) at a density of 1.5 × 10^5^ cells/well in 100 µL of DMEM/F-12 (without Phenol Red) and treated with 500 nM of either of the ApoE isoforms. After 30 min, some of the cells were exposed to Aβ1–42 at a concentration of 5 µM for various amounts of time (3 and 18 h) at 37 °C. After the 3 and 18 h incubation with or without Aβ1–42, Thioflavin T (ThT) was added to cells at a concentration of 15 µM, and ThT fluorescence was measured as described previously [16].

### 4.9. Scanning Electron Microscopy of Surface Attachment and Fibrillation of Aβ1–42

Differentiated WT and SDC3-overexpressing SH-SY5Y cells were grown on poly-D-lysine-coated glass-bottom 35 mm culture dishes. After 24 h, the cells were treated with ApoEs (at a concentration of 500 nM) in DMEM/F12 medium (supplemented with 10% FCS) at 37 °C. After 30 min, some of the cells were exposed to Aβ1–42 at a concentration of 5 μM. After 3 or 18 h of incubation with or without Aβ1–42, the cells were rinsed two times with ice-cold PBS, fixed in 2.5% glutaraldehyde and 0.15% alcian blue 8GX (Sigma, St. Louis, MO, USA) and progressed towards scanning electron microscopy (with a JEOL JSM-7100F/LV scanning electron microscope [JEOL SAS, Croissy-sur-Seine, France]) as described previously [16,19].

### 4.10. Statistical Analysis

Results are expressed as means ± standard error of the mean (SEM). Differences between experimental groups were evaluated using one-way analysis of variance (ANOVA). Values of *p* < 0.05 were accepted as significant. Pearson’s correlation coefficient was calculated for the correlation between the SDC3 or HS expression and the Aβ1–42 uptake and fibrillation values of SH-SY5Y cells. For colocalization analyses, the MOC and PCC were calculated as described previously [16,19].

## Data Availability

Data are contained within the article or Supplementary Materials.

## Supplementary Materials

The following are available online at https://www.mdpi.com/article/10.3390/ijms22137070/s1.
