# Supplementary material for: The Interplay of Apoes with Syndecans in Influencing Key Cellular Events of Amyloid Pathology
Source: Int J Mol Sci. 2021 Jun 30;22(13):7070. doi: 10.3390/ijms22137070 (PMC8268055; doi:10.3390/ijms22137070)
Supplement: Supplementary file 1 [file ijms-22-07070-s001.zip › ijms-1245499-supplementary.pdf]

Supplementary Materials

The Interplay of Apoes with Syndecans in Influencing Key Cellular Events of Amyloid Pathology

Anett Hudák, Katalin Jósvay, Ildikó Domonkos, Annamária Letoha, László Szilák and Tamás Letoha

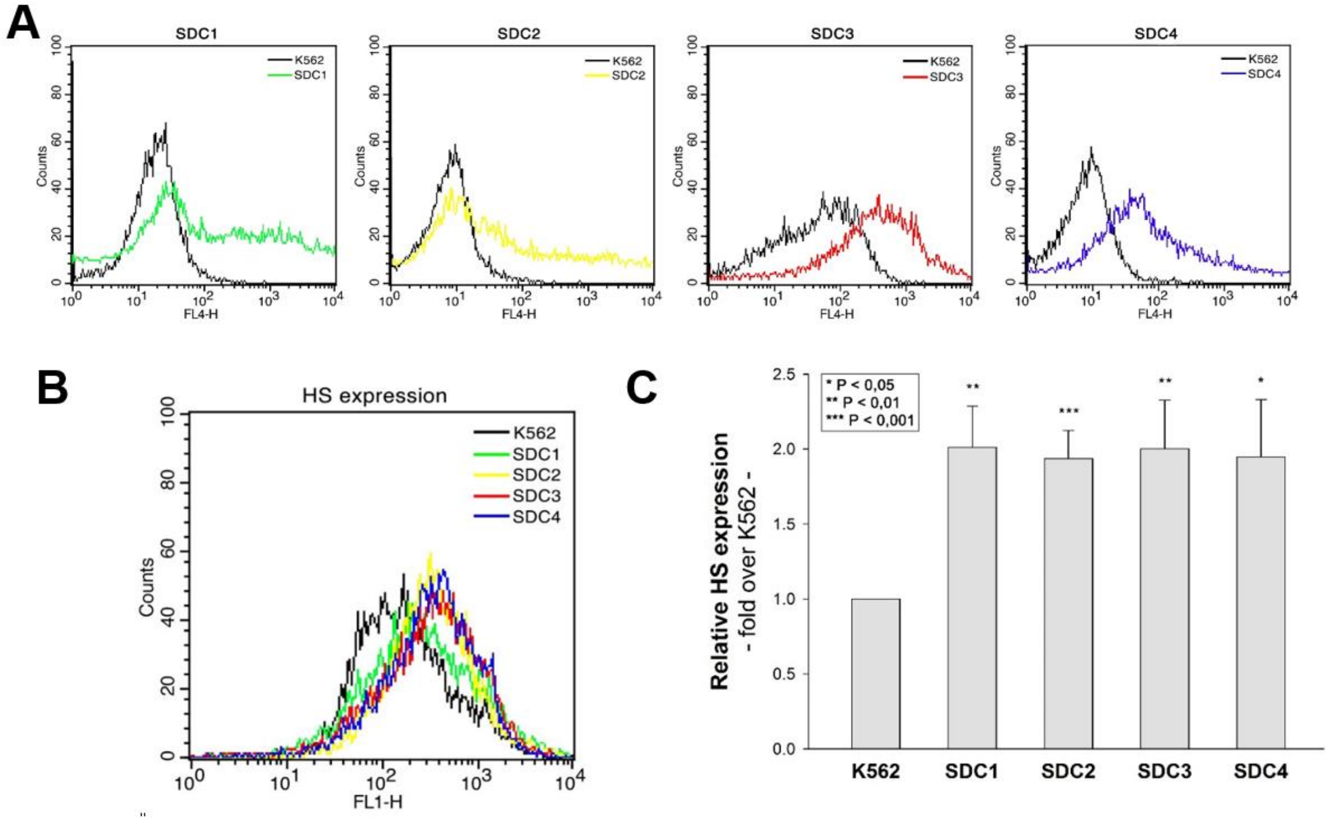

Figure S1. Relative HS expression of SDC transfectants. (**A**) Stable SDC transfectants created in wild-type (WT) K562 cells were selected by measuring SDC expression with flow cytometry (Becton Dickinson FACScan) using APC-labeled anti-SDC antibodies specific for each SDC isoform. (**B**) Flow cytometry histograms showing HS expression of SDC transfectants and WT K562 cells. HS expression of SDC transfectants, along with WT K562 cells, was measured with flow cytometry by using anti-HS antibody. SDC transfectants with a similar amount of HS expression were selected and applied for further studies. (**C**) Detected HS levels were normalized to WT K562 cells as standards. The bars represent the mean ± SEM of ten independent experiments. Statistical significance vs. WT K562 cells (standards) was assessed with analysis of variance (ANOVA). *$p < 0.05$; **$p < 0.01$, ***$p < 0.001$.

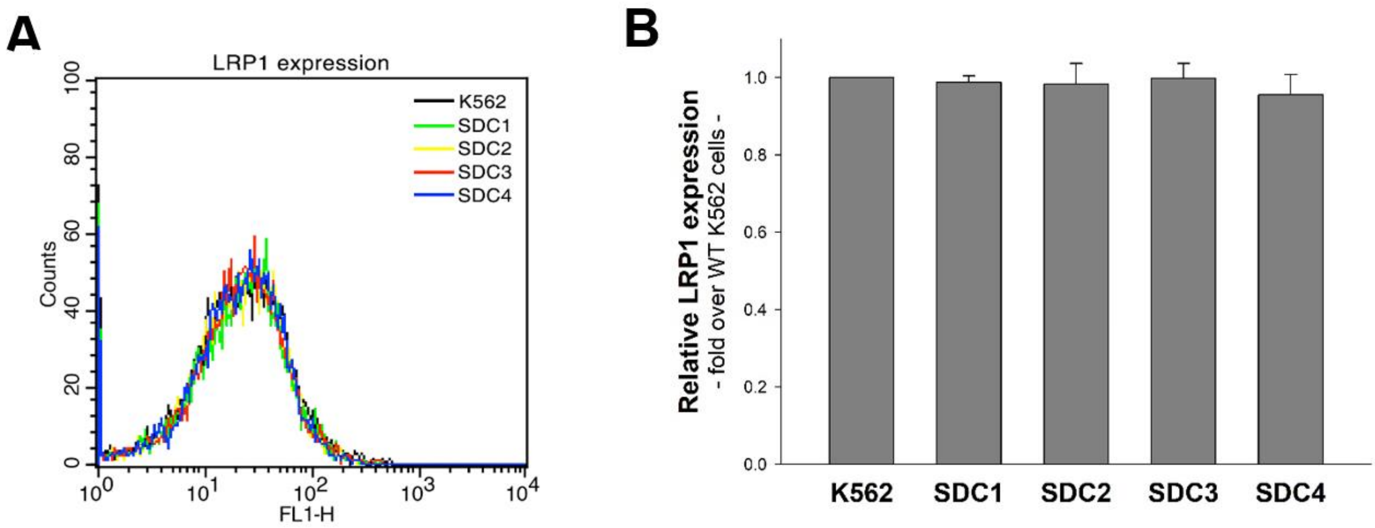

Figure S2. Relative LRP1 expression of K562 cells and SDC transfectants. **(A)** Flow cytometry histograms showing LRP1 expression of SDC transfectants and WT K562 cells using FITC-labeled anti-LRP1 antibody (cat.no.: sc-19616 FITC anti-LRP1 antibody, Santa Cruz) **(B)** Detected LRP1 levels were normalized to WT K562 cells as standards. The bars represent the mean ± SEM of three independent experiments. Statistical significance vs. WT K562 cells (standards) was assessed with ANOVA. Compared to WT K562 cells, no statistically significant differences were detected in LRP1 expression of SDC transfectants.

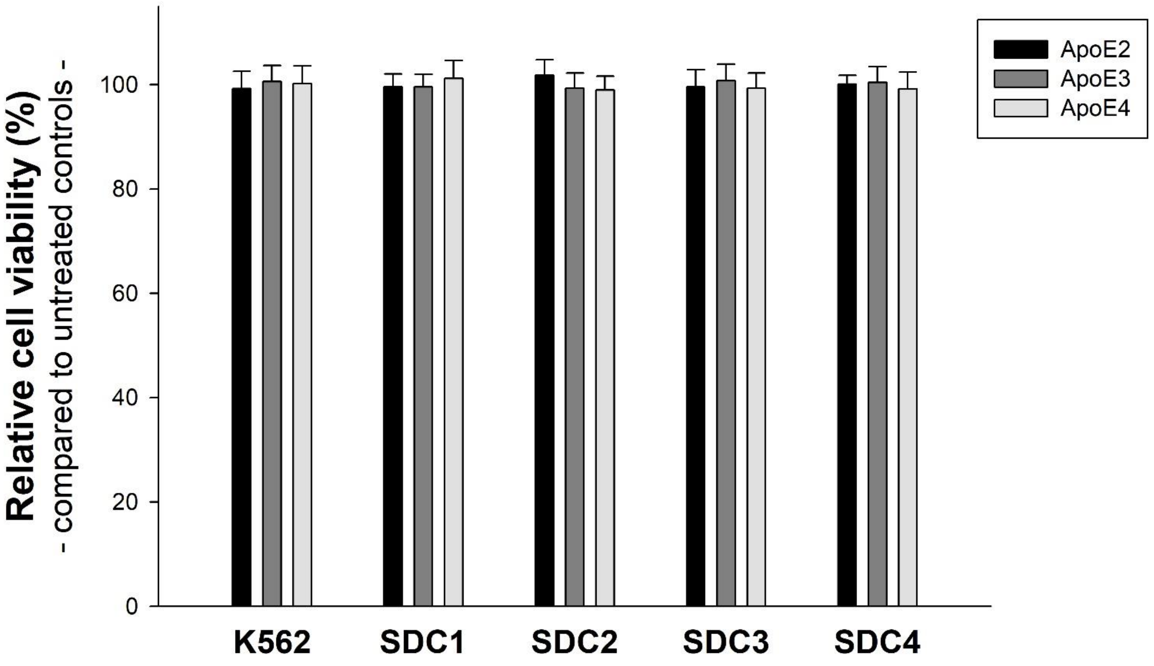

Figure S3. ApoEs does not affect cell viability during cellular uptake studies. Cellular uptake of ApoEs was measured by analyzing a minimum of 10,000 events per sample with flow cytometry. Viability of cells was determined by appropriate gating in a forward-scatter-against-side-scatter plot to exclude dead cells and debris. Thus percentage of live cells was measured. Detected percentage of live cells were normalized to ApoE-untreated cells (controls) as standards. The bars represent the mean ± SEM of four independent experiments. Statistical significance vs. standards was assessed with ANOVA. Compared to ApoE-untreated controls, no statistically significant differences were detected in the viability of ApoE-treated cells.

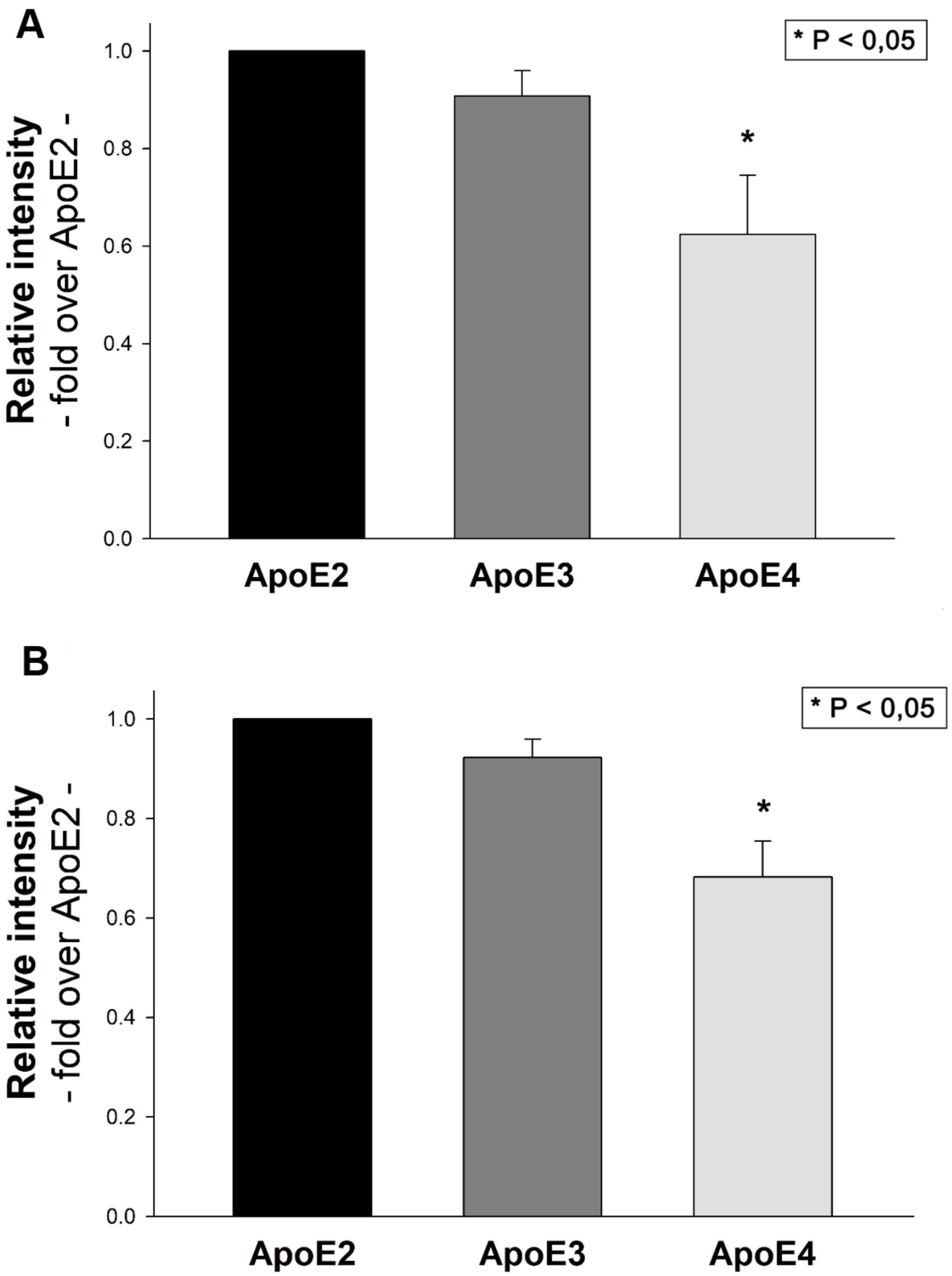

Figure S4. Quantification of SDS-PAGE bands of FITC-labeled ApoEs immunoprecipitated with SDC3 from extracts of SDC3 transfectants (**A**) or WT SH-SY5Y cells (**B**). The fluorescent signal of FITC-labeled ApoEs was detected with UVITEC Alliance Q9 Advanced Imager. The intensity of bands was analyzed with the NineAlliance© software. Detected band intensities were normalized to those of ApoE2 as standard. The bars represent the mean ± SEM of three independent experiments. Statistical significance vs. standards was assessed with ANOVA. *$p < 0.05$.

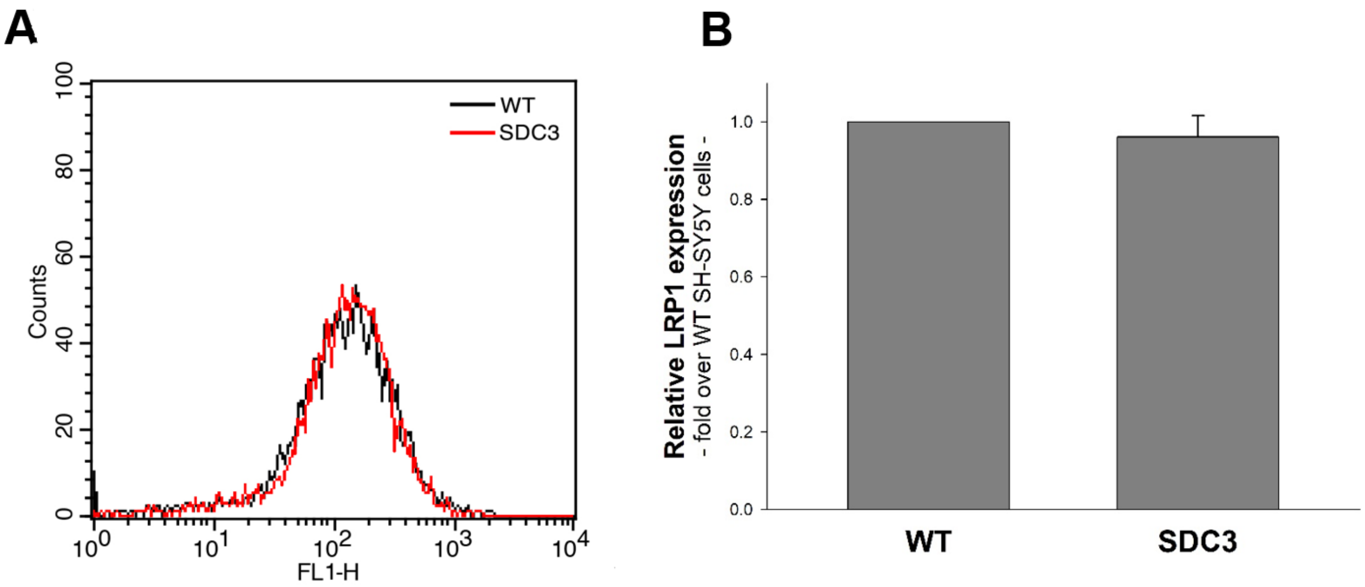

Figure S5. SDC3 overexpression does not affect LRP1 expression in SH-SY5Y cells. **(A)** Flow cytometry histograms showing LRP1 expression of WT and SDC3 overexpressing SH-SY5Y cells. **(B)** Detected LRP1 levels were normalized to WT SH-SY5Y cells as standards. The bars represent the mean ± SEM of three independent experiments. Statistical significance vs. standards was assessed with ANOVA. Compared to WT SH-SY5Y cells, no statistically significant differences were detected in LRP1 expression of SDC3 transfectants.

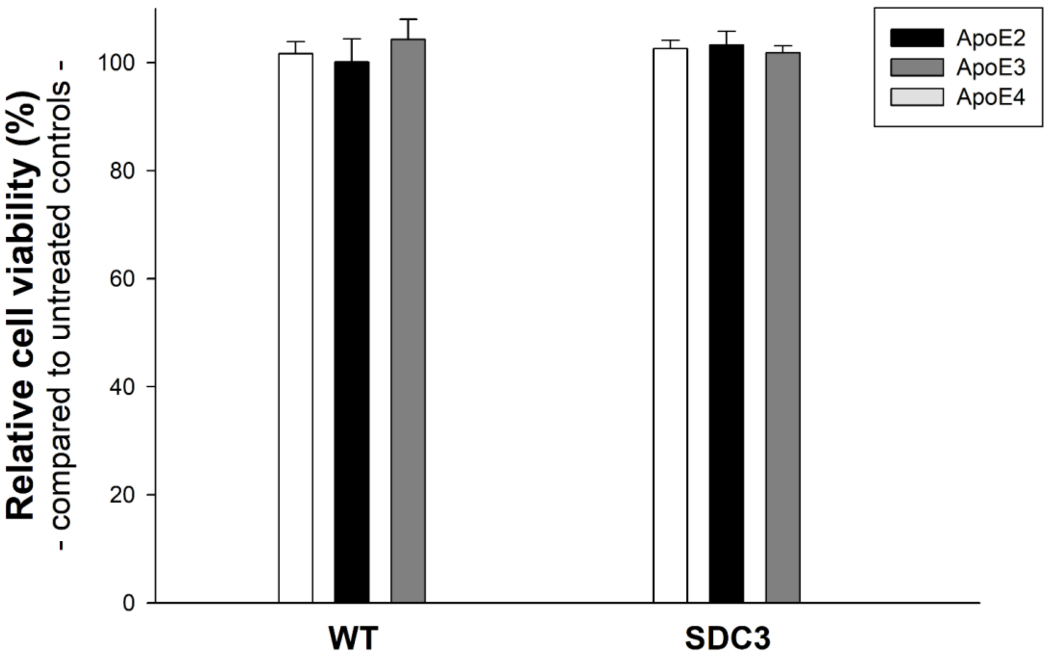

Figure S6. ApoEs do not affect cell viability during cellular uptake studies on WT and SDC3 overexpressing SH-SY5Y cells. Cellular uptake of ApoEs was measured by analyzing a minimum of 10,000 events per sample with flow cytometry. Viability of cells was determined by appropriate gating in a forward-scatter-against-side-scatter plot to exclude dead cells and debris. The detected percentage of live cells were normalized to ApoE-untreated cells as standards. The bars represent the mean ± SEM of four independent experiments. Statistical significance vs. standards was assessed with ANOVA. Compared to controls, no statistically significant differences were detected in the viability of ApoE-treated cells.

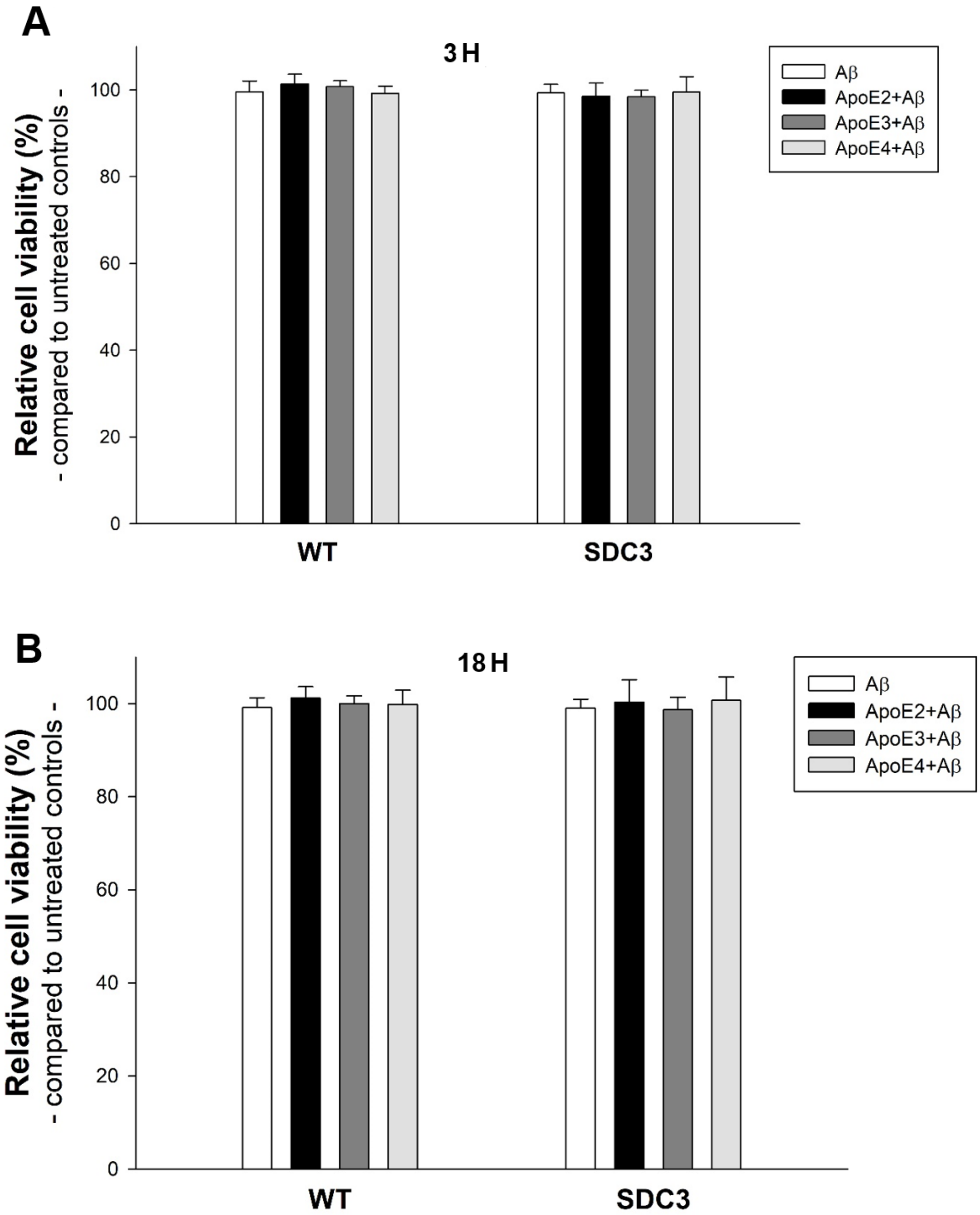

Figure S7. At the applied concentrations, Aβ1–42, either alone or preincubated with ApoEs, does not affect cell viability of WT and SDC3 overexpressing SH-SY5Y cells either after 3h (**A**) or 18 h (**B**) of incubation. Cellular uptake of Aβ1–42 was measured by analyzing a minimum of 10,000 events per sample with flow cytometry. Viability of cells was determined by appropriate gating in a forward-scatter-against-side-scatter plot to exclude dead cells and debris. (**A,B**) The detected percentage of live cells were normalized to Aβ1–42-untreated cells (i.e., controls) as standards. A: The bars represent the mean ± SEM of four independent experiments. Statistical significance vs. standards was assessed with ANOVA. Compared to untreated controls, no statistically significant differences were detected in viability of Aβ1–42-treated cells (co- or unincubated with ApoEs).

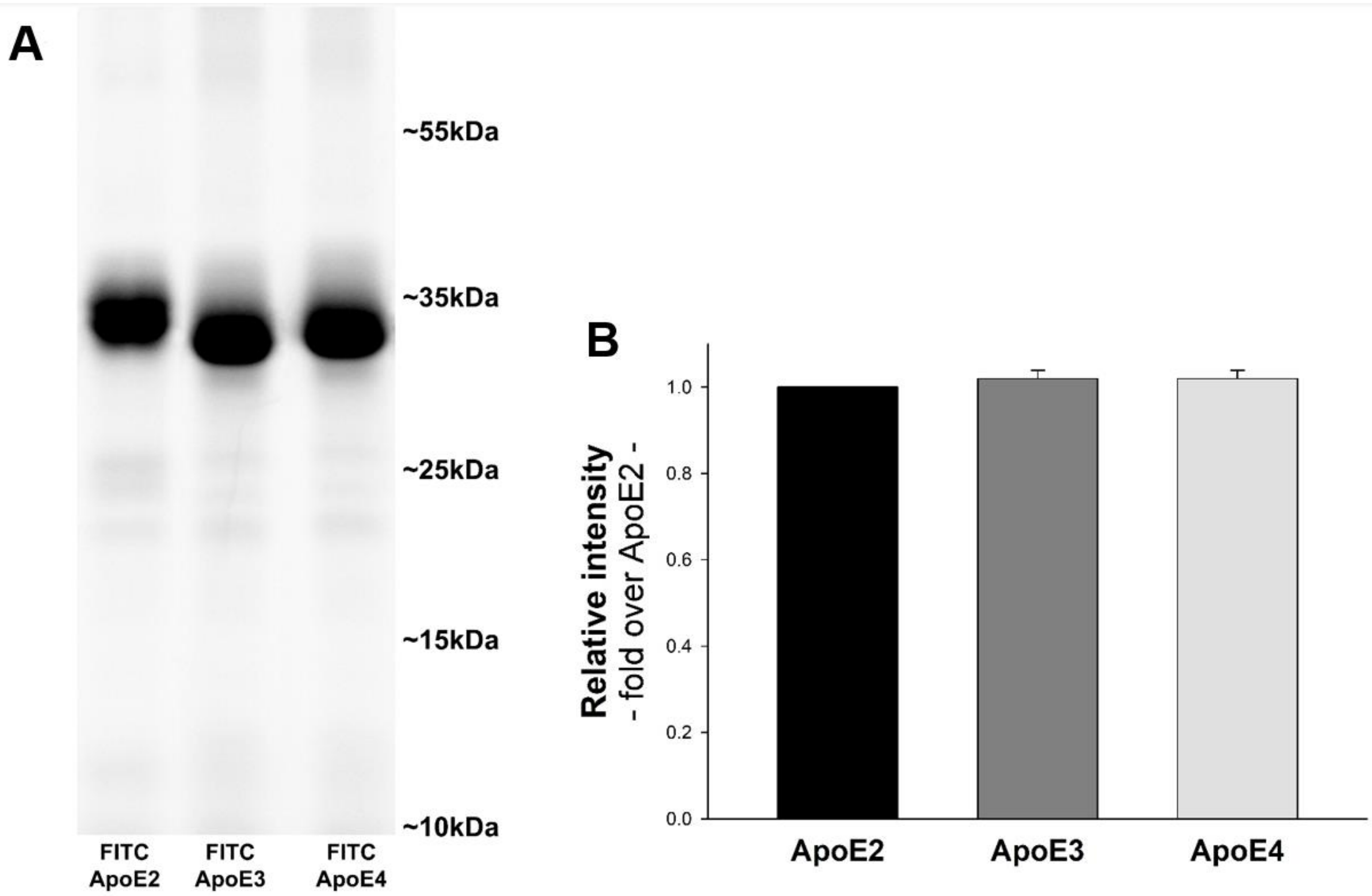

Figure S8. SDS-PAGE of the FITC-labeled ApoE isoforms. Fluorescent labeling of ApoE isoforms was performed with the Pierce™ FITC Labeling Kit according to the manufacturer's instructions. Protein concentrations were then measured with a spectrophotometer (Metertech UV/VIS). 10–10 µg of FITC-labeled ApoE isoforms were then subjected to SDS-PAGE. The fluorescent signal of FITC-labeled ApoEs was detected with UVITEC Alliance Q9 Advanced Imager, and the intensity of bands were analyzed with the NineAlliance© software. (**B**) Detected band intensities were normalized to ApoE2 as standard. The bars represent the mean ± SEM of three independent experiments. Statistical significance vs. standards was assessed with ANOVA. Compared to ApoE2, no statistically significant differences were detected.
